# Imbalanced mucosal microcirculation in the remission stage of ulcerative colitis using probe-based confocal laser endomicroscopy

**DOI:** 10.1186/s12876-019-1037-6

**Published:** 2019-07-01

**Authors:** Yu Tian, Yue Zheng, Guigen Teng, Junxia Li, Huahong Wang

**Affiliations:** Gastroenterology Department of Peking University First Hospital, Beijing, China

**Keywords:** Ulcerative colitis, Confocal laser Endomicroscopy, Microcirculation, Inflammatory bowel disease

## Abstract

**Background:**

Microcirculatory disturbance is an important factor in the pathogenesis of Inflammatory Bowel Disease (IBD) but there have been few studies in this field. Confocal Laser Endomicroscopy (CLE) has been used over the last 10 years and has made it possible to explore the changes in microcirculation of the colonic mucosa.

**Methods:**

We retrospectively selected patients who underwent probe-based Confocal Laser Endomicroscopy (*p*CLE) between 2014 and 2016. There were 7 patients with ulcerative colitis (UC) in clinical remission and 7 healthy subjects included in this study; all the UC patients’ medical data were reviewed. For each patient, three segments of the colon were examined using *p*CLE including the ascending, transverse/descending and sigmoid colon. In each segment, the representative *p*CLE images of the three sites were selected for analysis. Four indicators, including Mean Vessel Diameter (MVD), Diameter Standard Deviation (DSD), Functional Capillary Density-long (FCDL) and Functional Capillary Density-area (FCDA), were measured with a specially designed detection software algorithm. The four indicators were compared between UC patients and healthy subjects. According to the different blood flow patterns, three types of distribution were established: the Around (A), Cobweb (C) and Deficiency (D) type. The relationships between the recurrence and blood flow patterns of UC patients were analyzed.

**Results:**

MVD, DSD, FCDL and FCDA were 10.62 ± 0.56 μm, 2.23 ± 0.26, 0.030 ± 0.019 μm and 0.289 ± 0.030 for the healthy subjects and 11.06 ± 1.10 μm, 2.68 ± 0.29, 0.026 ± 0.005 μm and 0.272 ± 0.034 for the UC patients, respectively. Compared with healthy subjects, DSD was significantly increased and FCDA was significantly decreased (*P* < 0.01 for both). There was no difference in MVD and FCDL between UC patients and healthy subjects. The type A and type C blood flows were observed in healthy subjects (66.67 and 33.33%, respectively) while type C appears more in UC patients (71.3%) and type D blood flow could only be found in UC patients (14.29%) *P* < 0.01. UC patients who showed Type D blood flow had a shorter recurrence interval.

**Conclusions:**

Some local mucosal capillary density in UC patients was decreased, particularly in the inflammation-affected segment. The three mucosal blood flow patterns can be used as an indicator of mucosal healing.

## Background

Inflammatory bowel disease (IBD) is comprised of two forms of intestinal disorders: ulcerative colitis (UC) and Crohn’s disease (CD) [1]. Microcirculatory disturbance and angiogenesis are considered important components for the pathogenesis of IBD [2]. Many studies have shown that there is a close connection between chronic inflammation and angiogenesis. Hypoxia is regarded as the most important stimulator of these two conditions [3].

In the last decade, CLE has emerged as a new endoscopic imaging modality that enables real-time in vivo histologic examination [4]. Some studies have shown that CLE has the capability to perform a histopathological diagnosis of IBD, evaluate the disease status and predict the response to medical therapy [5]. Among the observed CLE image changes of UC patients, mucosal capillary blood flow is a factor deserving special attention and regarded as a unique advantage of CLE detection [6].

Dextran sulfate sodium (DSS)-induced colitis mice have been used to investigate the pathogenesis of UC for many years. Some research revealed that before epithelial cell damage appeared DSS administration elicited capillary vessel disruption. From this point of view mucosal microcirculatory disturbance became the triggers for DSS-induced colitis [7]. Similar phenomenon has been found in IBD patient, that is microcirculatory reorganization and extensive angiogenesis occurred in the inflamed sites [8]. The newly formed capillaries stimulated by chronic inflammation were relatively immature and hypoperfused. The ischemic conditions lead to additional inflammatory cell recruitment and sustained the inflammatory response [9].

The purpose of this study was to investigate the changes of colonic mucosal blood flow of UC patients in clinical remission and establish an easy-to-use classification for the mucosal microcirculation judgment of UC.

## Methods

### Research design

We conducted a retrospective study on patients who underwent *p*CLE between 2014 and 2016 in Peking University First Hospital. Because the study focused on the remission stage of UC, we selected patients with the following criteria:

1. The period of clinical remission was at least half a year;
2. Glucocorticoids had been stopped for at least 3 months;
3. The total Mayo score (clinical and endoscopic criteria) was ≤2, and the endoscopic score was Mayo 0 or 1.

A total of 11 UC patients underwent *p*CLE during the period and four of them were excluded because of either an active stage (3 cases) or incomplete data of *p*CLE examination (1 case). After the exclusion, seven UC patients were included. The healthy subjects underwent *p*CLE in the same period and showed no other disease in the examination.

All of the subjects (UC patients and healthy subjects) were voluntary and signed an informed consent before *p*CLE examination. In addition, the Clinical Trial Ethics Committee of Peking University First Hospital approved this study.

### *p*CLE imaging

The *p*CLE procedures were performed by experienced endoscopists with the subjects under standard sedation and contrasting with the intravenous application of 4 ml 10% fluorescein sodium and using the *p*CLE system (Cellvizio, ColoFlex™ UHD, Mauna Kea Technologies, France). The depth of the observation of ColoFlexTM UHD was between 55 and 65 μm [10], which is exactly the level that the mucosal blood flow could display [11]; the resolution was 1 μm with 1000-fold magnification in vivo in real time and the scan rate was 12 frames per second.

The *p*CLE images were obtained in three segments of UC patients and healthy subjects including ascending, transverse/descending (some sites could not be distinguished because of colonic shortening in some UC patients) and the sigmoid colon. In each segment of the colon, three sites were selected randomly to obtain the images. Clear *p*CLE images were recorded for at least 60 s in every site and valuable representative images are selected for analysis.

### Data processing

**Fig. 1:**
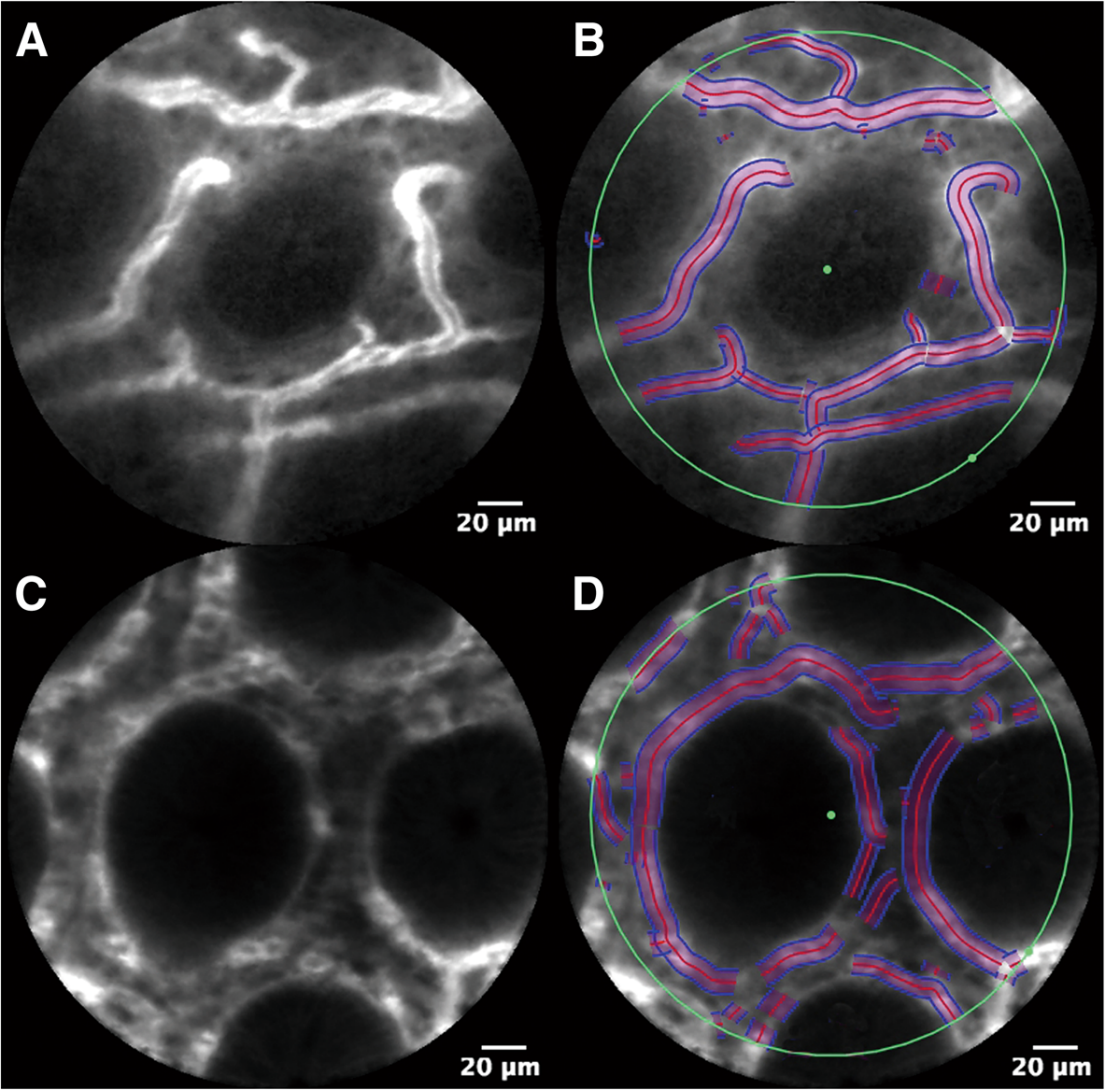
Mucosal blood flow recognition. The blood vessel recognition in the central region of the image was good and the analytical data were accurate and reliable. **a** and **c**: mucosal blood flow for a normal transverse colon and an unaffected segment of ulcerative colitis are shown; **b** and **d**: blood vessel recognition in the central region.

We selected 3 representative images from every site, which showed clear blood flow as the object image. To reduce the vascular recognition errors in the margins, the central area of the image was defined as the detection range. The IC Viewer software was used (VesselDetection, Mauna Kea Technologies, French) for analysis and to calculate the Mean Vessel Diameter (MVD), Diameter Standard Deviation (DSD), Functional Capillary Density-long (FCDL) and Functional Capillary Density-area (FCDA) of the mucosa [6]. When the blood vessel recognition was complete, the four indicators were automatically calculated by the software as shown in Fig. 1a-d.

**Fig. 2:**
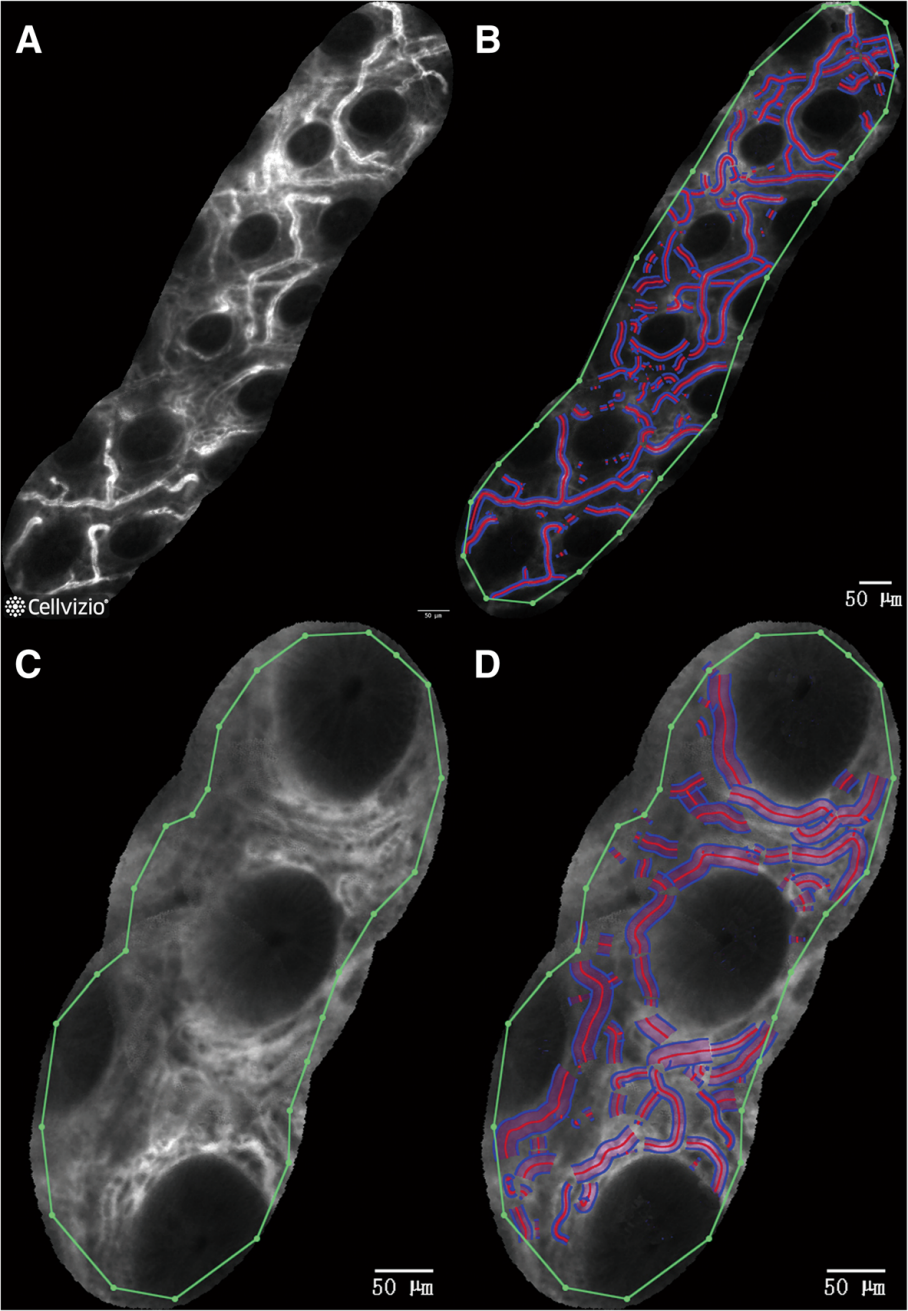
Mucosal blood flow shown by the processed rebuild image. **a** and **c** a normal transverse colon and an affected Mayo score 0 mucosal blood flow rebuild image are shown; **b** and **d**: blood vessel recognition within the limited range

To process a large surface, good image records were first analyzed with the software (IC Viewer MosaicMauna Kea Technologies, French) to perform mosaicking, which consists of stitching consecutive frames to rebuild the images [12]. Then, the previous procedure to calculate the four indicators was followed as shown in Fig. 2a-d.

### Mucosal blood flow patterns

According to the different summarized patterns, the blood flow distributions of UC patients and healthy subjects were analyzed. For each segment, the most serious type of blood flow was determined as the data of this segment. The ratio of different mucosal blood flow patterns between UC patients and healthy subjects was compared.

### A review of patients’ records

From 2014 to 2018, we reviewed the 7 UC patients’ medical records including visit records, prescriptions and hospitalization records. The longest time interval in the retrospective records was 3 months. The criteria for clinical recurrence included the following items:

1. Occurrence of obvious bloody stool accompanied by elevated C-reactive protein or erythrocyte sedimentation rate;
2. Clinical Mayo score (excluding the colonoscopic score) more than 5; and.
3. Colonoscopic Mayo score judged by colonoscopy≥2.

A relationship between clinical recurrence and mucosal blood flow morphology was observed and the main treatment adjustment was listed.

### Statistical analysis

The statistical analysis was performed using SAS software (version 9.4, SAS Institute Inc., Cary, NC, USA). Continuously measured parameters are presented as mean ± standard deviation, while categorical data are presented as the frequency with the proportion. Because of the unbalanced distribution of the data, we did not use the analysis of variance to compare means of each measured parameter. Instead, we used the general linear model (GLM procedure in SAS) to compare means among the affected statuses (four groups: control; unaffected segment; and affected segment Mayo score 0 and 1). Fisher’s exact test was used to compare the distribution of the proportions. A *P*-value < 0.05 was considered statistically significant.

## Results

### General data

Neither UC patients nor healthy subjects had previous cardiovascular disease or a history of diabetes; one UC patient had mild hypertension but their blood pressure was under control. The age of healthy subjects ranged from 36 to 59 with a median of 48 years old. The other data are shown in Table 1.

**Table 1:** Patient Characteristics

Patient Characteristics		
No. of Subjects		
UC patients	7	
Healthy subjects	7	
Median age (min, max), year		
UC patients	50	(22,75)
Healthy subjects	48	(36,59)
Sex, female	4/7	57.1%
Medical history, y	15.3	
Extent of disease		
E2	2/7	28.6%
E3	5/7	71.4%
Main treatment of the induction period		
5-ASA	1/7	14.3%
Glucocoricoid	4/7	57.1%
Anti-TNF	2/7	28.6%
Severity of disease		
Moderate	3	42.9%
Severe	4	57.1%
Maintenance period treatment		
5-ASA	5/7	71.4%
AZA	2/7	28.6%

*5-ASA* 5-aminosalicylic acid (Mesalamine), *AZA* Azathioprine

The extent of disease and distribution of mucosal Mayo score by endoscopy of the 7 UC patients is shown in Table 2.

**Table 2:** Extent of disease and the distribution of the mucosal Mayo score

Case	Ascending colon	Transverse/Descending colon	Sigmoid colon
E2	Unaffected	Unaffected	Affected Mayo score 1
E3	Affected Mayo score 0	Affected Mayo score 0	Affected Mayo score 1
E3	Unaffected	Affected Mayo score 1	Affected Mayo score 1
E3	Affected Mayo score 0	Affected Mayo score 0	Affected Mayo score 0
E2	Unaffected	Unaffected	Affected Mayo score 0
E3	Affected Mayo score 0	Affected Mayo score 1	Affected Mayo score 1
E3	Unaffected	Affected Mayo score 1	Affected Mayo score 1

### Comparison of mucosal blood flow between UC patients and healthy subjects

The MVD, DSD, FCDL and FCDA were 10.62 ± 0.56 μm, 2.23 ± 0.26, 0.030 ± 0.019 μm and 0.289 ± 0.030 for the healthy subjects and 11.06 ± 1.10 μm, 2.68 ± 0.29, 0.026 ± 0.005 μm and 0.272 ± 0.034 for the UC patients, respectively. In summary, the general linear model was used to test whether there was a statistical difference between UC patients of different affected statuses (unaffected, affected Mayo score 0, and affected Mayo score 1) and healthy subjects. The distribution of DSD between UC patients and healthy subjects showed a statistical difference (*P*<0.01), and the distribution of FCDA also showed a statistical difference (*P*<0.05). There was no statistical difference in the distribution of the other indicators between UC patients and healthy subjects. Because of the uneven data distribution, an additional comparison was not conducted for each subgroup as shown in Table 3.

**Table 3:** Comparison of the mucosal blood flow between UC patients and healthy subjects

Characteristics	MVD	DSD	FCDL	FCDA
All subjects are without distinguishing areas of the colon				
Healthy subjects (*n* = 21)	10.62 ± 0.56	2.23 ± 0.26	0.030 ± 0.019	0.289 ± 0.030
UC patients (*n* = 21)	11.06 ± 1.10	2.68 ± 0.29	0.025 ± 0.004	0.272 ± 0.034
UC-unaffected (*n* = 6)	11.01 ± 0.79	2.58 ± 0.25	0.025 ± 0.003	0.278 ± 0.026
UC-Mayo score 0 (*n* = 7)	10.98 ± 0.77	2.73 ± 0.25	0.027 ± 0.003	0.301 ± 0.017
UC-Mayo score 1 (*n* = 8)	11.17 ± 1.57	2.72 ± 0.36	0.022 ± 0.004	0.245 ± 0.031
*P*-value for affected status	0.29	< 0.001	0.76	0.005
Ascending colon				
Healthy subjects (*n* = 7)	10.95 ± 0.79	2.29 ± 0.30	0.039 ± 0.032	0.303 ± 0.026
UC patients (*n* = 7)	11.10 ± 0.89	2.81 ± 0.20	0.029 ± 0.004	0.290 ± 0.032
UC-unaffected (*n* = 4)	11.02 ± 1.02	2.67 ± 0.13	0.028 ± 0.005	0.275 ± 0.033
UC-Mayo score 0 (*n* = 3)	11.21 ± 0.89	2.99 ± 0.11	0.03^a^	0.310 ± 0.020
Transverse/Descending colon				
Healthy subjects (*n* = 7)	10.55 ± 0.39	2.12 ± 0.16	0.024 ± 0.005	0.267 ± 0.031
UC patients (*n* = 7)	11.26 ± 1.56	2.52 ± 0.40	0.026 ± 0.005	0.276 ± 0.022
UC-unaffected (*n* = 2)	11.00 ± 0.14	2.39 ± 0.40	0.03^a^	0.28^a^
UC-Mayo score 0 (*n* = 2)	10.65 ± 1.20	2.55 ± 0.07	0.025 ± 0.007	0.300 ± 0.014
UC-Mayo score 1 (*n* = 3)	11.84 ± 2.38	2.59 ± 0.60	0.023 ± 0.006	0.257 ± 0.015
Sigmoid colon				
Healthy subjects (*n* = 7)	10.36 ± 0.28	2.28 ± 0.29	0.029 ± 0.004	0.297 ± 0.020
UC patients (*n* = 7)	10.82 ± 0.82	2.72 ± 0.19	0.024 ± 0.005	0.251 ± 0.037
UC-Mayo score 0 (*n* = 2)	10.97 ± 0.37	2.53^a^	0.03^a^	0.285 ± 0.007
UC-Mayo score 1 (*n* = 5)	10.77 ± 0.97	2.80 ± 0.17	0.022 ± 0.004	0.238 ± 0.036

Note: ^a^Only the mean value was presented if no variance existed

A *P* < 0.05 was considered statistically significant

### Mucosal blood flow morphology

**Fig. 3:**
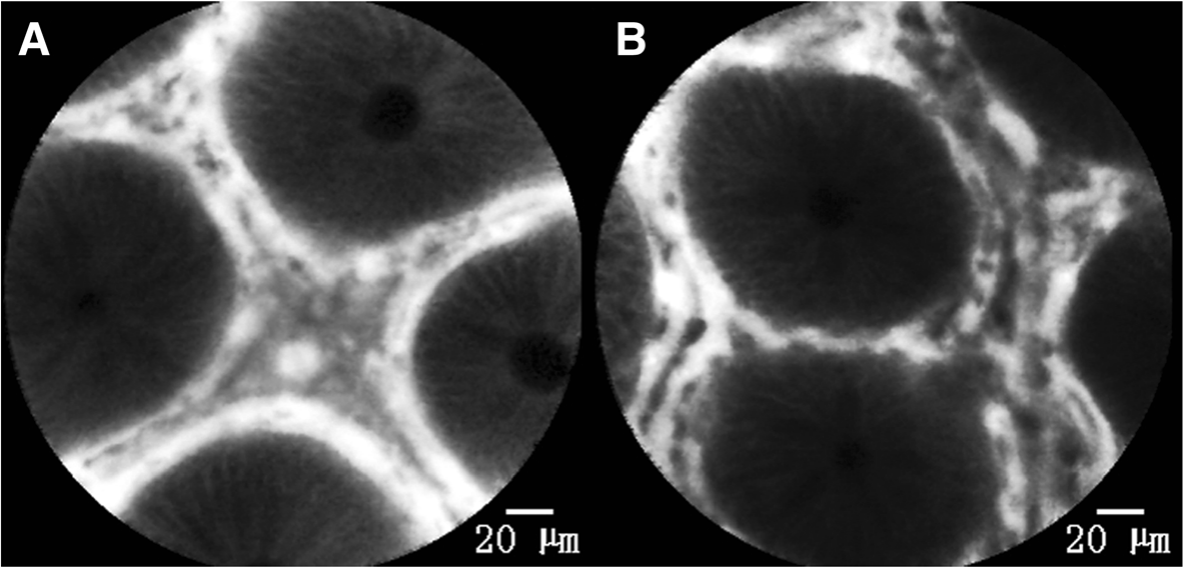
Type A of mucosal blood flow. **a** and **b** can be classified into type A blood flow. **a** shows a typical type A blood flow and the gland is regularly arranged. The capillaries in the lamina propria interstitial space are located around the gland. This type of blood flow is mainly distributed in the transverse and descending colon of healthy people. In **b**, the number of capillaries is slightly increased and the width of the lamina propria interstitial space is slightly different. The proliferating capillaries are still parallel to other blood flow and along the glands. This type of blood flow is distributed in healthy subjects and is also found in the unaffected segments of UC patients

**Fig. 4:**
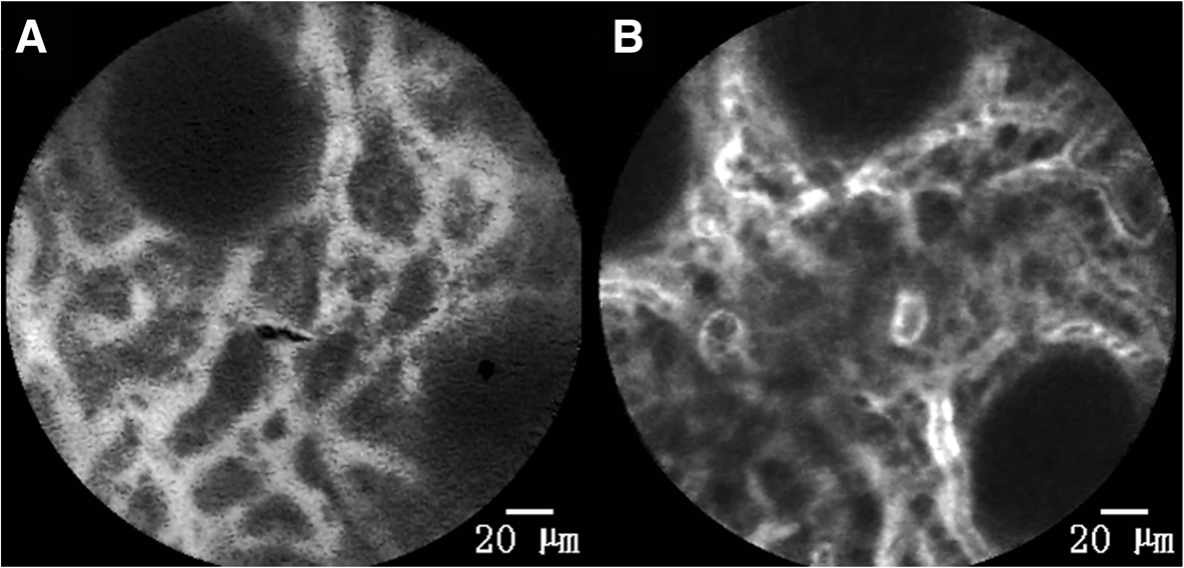
Type C of mucosal blood flow. **a** and **b** show type C blood flow. This type of blood flow distribution is characterized by an obviously wide lamina propria interstitial space and blood flow crisscrossing into a network structure. In **a**, the fluorescence intensity of blood flow was homogeneous and reflects good capillary filling and a homogeneous distribution of blood flow. This is mainly seen in the unaffected segments and affected Mayo score 0 segments of UC patients and it is also found in the normal cecum and sigmoid colon. In **b**, the distribution of blood flow is more irregular and the distribution of fluorescein is uneven. This reflects a lack of blood flow in some parts of the capillaries. This type of blood flow is mainly distributed in the affected Mayo score 0 segments of UC patients

**Fig. 5:**
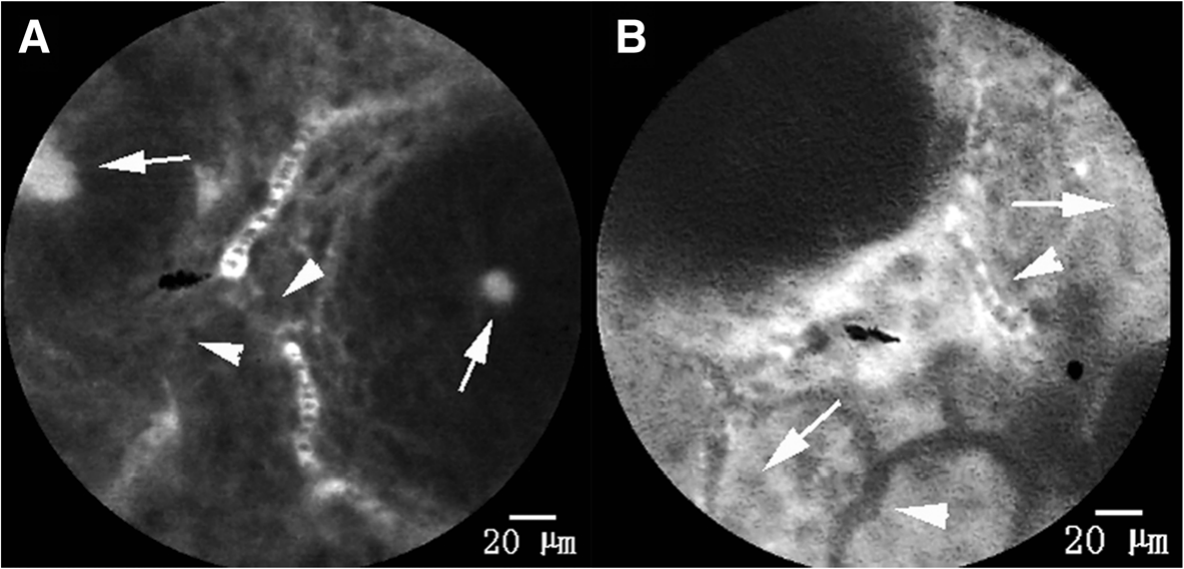
Type D of mucosal blood flow. **a** and **b** are type D blood flow, which only occurs in the affected Mayo score 1 segments of UC patients. **a** shows a blood flow that is squeezed and interrupted by many inflammatory cells that have infiltrated into the lamina propria interstitial space (as indicated by the arrowhead) and is accompanied by a leakage of fluorescein in the glandular tube (as indicated by the arrow). **b** shows that there is a large amount of fluorescein leakage in the lamina propria interstitial space; in this background, we can see some capillaries with no blood flow (as indicated by the arrowhead), while in the others the fluorescein is fully filled (as indicated by the arrow). That is, blood flow is obviously uneven and “short circuit” blood flow emerges

The blood flow patterns of the colonic mucosa in healthy subjects and UC patients in remission were observed and classified into three groups: Around type (type A), Cobweb type (type C) and Deficient type (type D) as shown in Figs. 3, 4 and 5.

The distribution of blood flow patterns between affected status and the position of the colon were compared. Three sampling types of vascular distribution at the same segment may be inconsistent (for example, two As and one C), and the more serious type determined the data of this segment (type C). Most patients did not have type D and no corresponding proportion was given at this time (NA). The difference of blood flow pattern distribution was obvious between UC patients and healthy subjects (*P*<0.01). Further comparison showed that the difference of the blood flow distribution of UC patients and healthy subjects in the ascending and transverse/descending colon was also significantly different (*P*<0.05 and *P*<0.01, respectively).

Type A blood flow was the most common type of capillary distribution for healthy subjects and accounted for 66.67% of the data. Type C blood flow could be seen in healthy subjects and UC patients. Type D blood flow only appeared in UC patients of Mayo score 1 as shown in Table 4.

**Table 4:** Distribution of blood flow patterns in different segments of healthy subjects and UC patients

Characteristics	Type A	Type C	Type D	*P*-value for Fisher’s exact test
All subjects without distinguishing areas of the colon				
Healthy subjects (*n* = 21)	14(66.67%)	7(33.33%)	0(0%)	0.001
UC patients (*n* = 21)	3(14.29%)	15(71.43%)	3(14.29%)	
Ascending colon				
Healthy subjects (*n* = 7)	6(85.71%)	1(14.29%)	NA	0.03
UC patients (*n* = 7)	1(14.29%)	6(85.71%)	NA	
UC-unaffected (*n* = 4)	1(25%)	3(75%)	NA	1.00
UC-Mayo score 0 (*n* = 3)	0(0%)	3(100%)	NA	
Transverse/Descending colon				
Healthy subjects (*n* = 7)	7(100%)	0(0%)	NA	0.005
UC patients (*n* = 7)	1(14.29%)	6(85.71%)	NA	
UC-unaffected (*n* = 2)	1(50%)	1(50%)	NA	0.57
UC-Mayo score 0 (*n* = 2)	0(0%)	2(100%)	NA	
UC-Mayo score 1 (*n* = 3)	0(0%)	3(100%)	NA	
Sigmoid colon				
Healthy subjects (*n* = 7)	1(14.29%)	6(85.71%)	0(0%)	0.19
UC patients (*n* = 7)	1(14.29%)	3(42.86%)	3(42.86%)	
UC-Mayo score 0 (*n* = 2)	1(50%)	1(50%)	0(0%)	0.57
UC-Mayo score 1 (*n* = 5)	0(0%)	2(40%)	3(60%)	

### Patients’ records review and recurrence

Over nearly 5 years, all of the patients had clinical relapse and the minimum was at least one time, while the maximum was 4 times of moderate or severe clinical recurrence. There were moderate to more severe relapses in three patients with type D blood flow within 1 year. Among the three patients with type D blood flow, there were two patients that received a step-up treatment scheme as shown in Table 5.

**Table 5:** Review and recurrence of patients’ records

UC patients	Age	Medical history (y)	Extent of the disease	Type D blood flow	Recurrent interval (m)	Maintenance therapy	Treatment changes (Y/N)
1	50	12	E2	N	13.7	5-ASA	N
2	35	15	E3	N	21	5-ASA	N
3	65	35	E3	Y	7.3	5-ASA	Y, Pred
4	45	5	E3	N	21	AZA	N
5	75	5	E2	N	22	5-ASA	N
6	67	32	E3	Y	9.5	5-ASA	N
7	22	3	E3	Y	9.7	AZA	Y, anti-TNF

*N/Y* yes or no, *y* years, *m* months, *5-ASA* 5-aminosalicylic acid (Mesalamine), *AZA* Azathioprine, *Pred* prednisone, *anti-TNF* tumor necrosis factor monoclonal antibody (Remicade)

## Discussion

The most interesting finding of this study is the imbalanced mucosal microcirculation, especially in the Mayo one score area of the affected segment. The phenomenon of local mucosal ischemia is correlated with incomplete mucosal healing in the remission of UC and a greater chance of recurrence. These results are different from certain previous studies, including enhanced CT and MR [13], Dynamic Contrast-enhanced Ultrasound [14], magnifying endoscopy and virtual chromoendoscopy (i.e., narrow band imaging) [15], which mostly found that mucosal blood flow was increased in the affected segment of active IBD patients and recovered to normal when they entered the remission period. The above studies were conducted from a macroscopic perspective. It is our speculation that although the whole mucosal blood flow increased because of the destruction of mucosal glands and inflammatory cell infiltration in the lamina propria, the lamina propria interstitial space was relatively increased [16]. In addition, in a local area observation using microscopy in some sites of lamina propria, the capillary density was decreased.

Four indicators, MCV, DSD, FCDL and FCDA, are interrelated, reflecting the changes of mucosal microcirculation, FCDL and FCDA are direct indicators. DSD represents the uniformity of capillary diameter. When DSD does not increase, the capillary diameter is uniform, FCDL and FCDA can both reflect the changes of mucosal microcirculation which always caused by vasodilatory capacity changes. The trends of the two indicators and MCV are consistent. When DSD increases, only FCDA can accurately reflect the changes of microcirculation. In this study, the changes of DSD and FCDA are related to the destruction, regeneration and remodeling of capillaries caused by inflammation. According to the study data, we can speculate that although the mucosal capillaries were compensatory changed (DSD increased), the decreased FCDA indicated local ischemia in UC patients, especially in inflammation-affected areas. There are some studies supporting the local imbalanced microcirculation theory [17, 18]. According to previous studies, some researchers examined the vasodilatory capacity of human intestinal micro vessels by measuring the in vitro vasodilatory response to acetylcholine from pressurized submucosal intestinal arterioles, which were rapidly isolated from intestinal specimens. Furthermore, chronically inflamed IBD arterioles showed a diminished vasodilatory capacity [19]. Thus, the microcirculation blood supply could not match the state of the oxygen demand and an intestinal injury appeared. Another study demonstrated the diminished capacity of colonic arterioles in response to endogenous endothelium and there was vascular dysfunction in the capillary remodeling [20]. Therefore, we postulated that severe inflammation is characterized by increased vascular perfusion in the early stage of the disease, while reduced regional blood flow is commonly seen in remodeled tissues in chronically inflamed period [17].

Furthermore, we tried to simplify and visualize the differences of mucosal microcirculation by mucosal blood flow morphological classification instead of measurement. Based on previous studies [21], three types of blood vessels were summarized in our study: Around type (type A), Cobweb type (type C) and Deficiency type (type D). Among healthy people, type A is the most common type and type C is relatively less distributed and is mainly found in the sigmoid colon and ascending colon. Compared with healthy subjects, UC patients showed vascular remodeling, especially in the inflammation of the affected segment, which expressed that the distribution of C blood flow increased significantly with obvious uneven fluorescence intensity. Type D blood flow could only be found in the inflammation affected Mayo one score segment in our study. This reflected local ischemia or imbalanced blood flow. Imbalanced blood flow could be caused by inflammatory cell infiltration and compression, by an exiting prothrombotic condition, or by emergence of “short circuit” blood flow [17]. In our study, such type D blood flow is often accompanied by various leakages of fluorescein.

The main limitation of this study is insufficient data. Although there were statistical differences between UC patients and healthy subjects, the comparison between subgroups could not be conducted because of insufficient data. Further studies with sufficient data will be able to discover the differences of mucosal blood flow among different statuses of the affected segment for UC. This can further reveal the characteristics of focal microcirculation disorders. Another main limitation stems from its retrospective nature. This might cause bias in patient selection; in addition, data collection might not be complete. Therefore, it is important to design a prospective study to explore the relationship between mucosal blood flow and recurrence. We hope that treatment by regulating local mucosal blood flow might become a promising therapy for UC maintenance in the future.

## Conclusion

Our findings suggest that colonic mucosal vasodilation in UC patients does not compensate for the need of blood supply, which leads to local ischemia. Microcirculatory indicators, especially for DSD and FCDA measured by *p*CLE, will become objective indicators for further investigation of UC pathogenesis. Three types of mucosal blood flow patterns will become indicators of mucosal healing for UC.

## Data Availability

The data generated or analyzed during the study are not publicly available due to the patients’ confidentiality. However, all of the data and images of this study are available from the corresponding author upon reasonable request.

## Abbreviations

5-ASA: 5-aminosalicylic acid
AZA: Azathioprine
CD: Crohn’s disease
CLE: Confocal Laser Endomicroscopy
CT: Computed Tomography
DSD: Diameter Standard Deviation
DSS: Dextran sulfate sodium
FCDA: Functional Capillary Density-area
FCDL: Functional Capillary Density-long
IBD: Inflammatory Bowel Disease
MR: Magnetic Resonance
MVD: Mean Vessel Diameter
*p*CLE: Probe-based Confocal Laser Endomicroscopy
UC: Ulcerative colitis

## References

[1] Ramos GP, Papadakis KA (2019). Mechanisms of disease: inflammatory bowel diseases. Mayo Clin Proc.

[2] Alkim C, Alkim H, Koksal AR, Boga S, Sen I (2015). Angiogenesis in inflammatory bowel disease. Int J Inflam.

[3] Pousa ID, Maté J, Gisbert JP (2008). Angiogenesis in inflammatory bowel disease. Eur J Clin Investig.

[4] Neumann H, Vieth M, Atreya R, Grauer M, Siebler J, Bernatik T, Neurath MF, Mudter J (2012). Assessment of Crohn's disease activity by confocal laser endomicroscopy. Inflamm Bowel Dis.

[5] Karstensen JG, Săftoiu A, Brynskov J, Hendel J, Ciocalteu A, Klausen P, Klausen TW, Riis LB, Vilmann P (2016). Confocal laser endomicroscopy in ulcerative colitis: a longitudinal study of endomicroscopic changes and response to medical therapy (with videos). Gastrointest Endosc.

[6] Schmidt C, Lautenschläger C, Petzold B, Sakr Y, Marx G, Stallmach A (2013). Confocal laser endomicroscopy reliably detects sepsis-related and treatment-associated changes in intestinal mucosal microcirculation. Br J Anaesth.

[7] Saijo H, Tatsumi N, Arihiro S, Kato T, Okabe M, Tajiri H, Hashimoto H (2015). Microangiopathy triggers, and inducible nitric oxide synthase exacerbates dextran sulfate sodium-induced colitis. Lab Investig.

[8] Koutroubakis IE, Tsiolakidou G, Karmiris K, Kouroumalis EA (2006). Role of angiogenesis in inflammatory bowel disease. Inflamm Bowel Dis.

[9] D'Alessio S, Tacconi C, Fiocchi C, Danese S (2013). Advances in therapeutic interventions targeting the vascular and lymphatic endothelium in inflammatory bowel disease. Curr Opin Gastroenterol.

[10] Nakai Y, Isayama H, Shinoura S, Iwashita T, Samarasena JB, Chang KJ, Koike K (2014). Confocal laser endomicroscopy in gastrointestinal and pancreatobiliary diseases. Dig Endosc.

[11] Gheonea DI, Saftoiu A, Ciurea T, Popescu C, Georgescu CV, Malos A (2010). Confocal laser endomicroscopy of the colon. J Gastrointestin Liver Dis.

[12] Quénéhervé L, David G, Bourreille A, Hardouin JB, Rahmi G, Neunlist M, Brégeon J, Coron E (2019). Quantitative assessment of mucosal architecture using computer-based analysis of confocal laser endomicroscopy in inflammatory bowel diseases. Gastrointest Endosc.

[13] Taylor SA, Punwani S, Rodriguez-Justo M, Bainbridge A, Greenhalgh R, De Vita E, Forbes A, Cohen R, Windsor A, Obichere A (2009). Mural Crohn disease: correlation of dynamic contrast-enhanced MR imaging findings with angiogenesis and inflammation at histologic examination--pilot study. Radiology..

[14] Saevik F, Nylund K, Hausken T, Ødegaard S, Gilja OH (2014). Bowel perfusion measured with dynamic contrast-enhanced ultrasound predicts treatment outcome in patients with Crohn's disease. Inflamm Bowel Dis.

[15] Danese S, Fiorino G, Angelucci E, Vetrano S, Pagano N, Rando G, Spinelli A, Malesci A, Repici A (2010). Narrow-band imaging endoscopy to assess mucosal angiogenesis in inflammatory bowel disease: a pilot study. World J Gastroenterol.

[16] Li CQ, Xie XJ, Yu T, Gu XM, Zuo XL, Zhou CJ, Huang WQ, Chen H, Li YQ (2010). Classification of inflammation activity in ulcerative colitis by confocal laser endomicroscopy. Am J Gastroenterol.

[17] Deban L, Correale C, Vetrano S, Malesci A, Danese S (2008). Multiple pathogenic roles of microvasculature in inflammatory bowel disease: a Jack of all trades. Am J Pathol.

[18] Harris NR, Carter PR, Watts MN, Zhang S, Kosloski-Davidson M, Grisham MB (2011). Relationship among circulating leukocytes, platelets, and microvascular responses during induction of chronic colitis. Pathophysiology..

[19] Hatoum OA, Binion DG, Otterson MF, Gutterman DD (2003). Acquired micro-vascular dysfunction in inflammatory bowel disease: loss of nitric oxide-mediated vasodilation. Gastroenterology.

[20] Mori M, Stokes KY, Vowinkel T, Watanabe N, Elrod JW, Harris NR, Lefer DJ, Hibi T, Granger DN (2005). Colonic blood flow responses in experimental colitis: time course and underlying mechanisms. Am J Physiol Gastrointest Liver Physiol.

[21] Hundorfean G, Chiriac MT, Mihai S, Hartmann A, Mudter J, Neurath MF (2017). Development and validation of a confocal laser Endomicroscopy-based score for in vivo assessment of mucosal healing in ulcerative colitis patients. Inflamm Bowel Dis.

